# Supplementary material for: Content-rich biological network constructed by mining PubMed abstracts
Source: BMC Bioinformatics. 2004 Oct 8;5:147. doi: 10.1186/1471-2105-5-147 (PMC528731; doi:10.1186/1471-2105-5-147)
Supplement: Additional File 2 — The original results of the above study (non-essential files are deleted to keep the file size under the limit set by BMC bioinformatics). [file 1471-2105-5-147-S2.bz2 › chilibotAdditionalFile2/dip10/349ID8910586E4497/html/IL13_IL13RA1.html]

 


 **IL13** and **IL13RA1** 
  
Found 9 abstracts in PubMed, retrieved 9.  
 

 What does Google say? 
 PDF only 
| .edu only 

---

**Interactive relationship** (e.g. stimulation, inhibition, etc)

**Inhibitory relationship**- However, treatment of IL 4 deficient mice with IL 13Ra  [ **IL13RA1** ]  during vvGs immunization reduced IL 5, IL 13  [ **IL13** ] , and eotaxin production and pulmonary eosinophilia after RSV challenge.  Ref: 12574374 J Immunol, 2003
- These findings suggest that different functional responses to IL 4 and IL 13  [ **IL13** ]  by human monocytes and macrophages may result from reduced expression of gamma c and IL 13R alpha1  [ **IL13RA1** ] .  Ref: 10534111 J Leukoc Biol, 1999
**Neutral relationship**- These results suggest that the IL 13  [ **IL13** ]  receptor alpha chain NR4  [ **IL13RA1** ]  is the primary binding subunit of the IL 13  [ **IL13** ]  receptor and may also be a component of IL 4 receptors.  Ref: 8552669 Proc Natl Acad Sci U S A, 1996
- In fact, IL 4R alpha with IL 13R alpha1  [ **IL13RA1** ]  is designated both as an IL 13  [ **IL13** ]  receptor and the type II IL 4 receptor.  Ref: 10534111 J Leukoc Biol, 1999
- By using transient expression in COS 7 cells, NR4  [ **IL13RA1** ]  was found to encode the IL 13  [ **IL13** ]  receptor alpha chain, a low affinity receptor capable of binding IL 13  [ **IL13** ]  but not IL 4 or interleukins, 9, or 15.  Ref: 8552669 Proc Natl Acad Sci U S A, 1996
- Analysis of cell lines Daudi and THP 1 that differentially express gamma c and IL 13R alpha1  [ **IL13RA1** ]  showed that IL 13  [ **IL13** ]  can activate STAT6 in IL 13R alpha1  [ **IL13RA1** ]  positive THP 1 cells but not in gamma c positive, IL 13R alpha1  [ **IL13RA1** ]  negative Daudi cells.  Ref: 10427971 Eur J Immunol, 1999
- Electrophoretic mobility shift assay showed that IL 13  [ **IL13** ]  and IL 4 were able to activate Stat6 in cells expressing both IL 4R alpha and IL 13R alpha1  [ **IL13RA1** ] , while no activation was observed in cells expressing either one or the other alone.  Ref: 9013879 FEBS Lett, 1997
- Stable expression of the IL 13  [ **IL13** ]  receptor alpha chain NR4  [ **IL13RA1** ]  in CTLL 2 cells resulted in the generation of high affinity IL 13  [ **IL13** ]  receptors capable of transducing a proliferative signal in response to IL 13  [ **IL13** ]  and, moreover, led to competitive cross reactivity in the binding of IL 4 and IL 13  [ **IL13** ] .  Ref: 8552669 Proc Natl Acad Sci U S A, 1996
- The primary IL 13  [ **IL13** ]  receptor complex on human monocytes is believed to be a heterodimer comprised of the IL 4R alpha chain and the IL 2R gamma chain gamma c like molecule, IL 13R alpha1  [ **IL13RA1** ] .  Ref: 10427971 Eur J Immunol, 1999

**Non-interactive relationship** (e.g. studied together, co-existance, homology, etc.)

- In the absence of functional IL 13R alpha1  [ **IL13RA1** ]  and gamma c, IL 13  [ **IL13** ]  must signal through an alternative receptor complex on MDMac.  Ref: 10427971 Eur J Immunol, 1999
- Cloning of the human IL 13R alpha1  [ **IL13RA1** ]  chain and reconstitution with the IL4R alpha of a functional IL 4 IL 13  [ **IL13** ]  receptor complex.  Ref: 9013879 FEBS Lett, 1997
- We hypothesized that variants in the  **IL13** ,  **IL13RA1** , and IL4RA genes would be associated with an accelerated rate of decline of lung function among smokers.  Ref: 12594065 Am J Respir Cell Mol Biol, 2003
- However, with reduced IL 13R alpha1  [ **IL13RA1** ]  expression and low nuclear STAT6 activity, some IL 13  [ **IL13** ]  induced responses were unaltered in magnitude in MDMac.  Ref: 10427971 Eur J Immunol, 1999
- The human homologue of the recently cloned murine IL 13  [ **IL13** ]  binding protein IL 13R alpha1  [ **IL13RA1** ]  was cloned from a cDNA library derived from the carcinoma cell line CAKI 1.  Ref: 9013879 FEBS Lett, 1997
- mRNA levels for IL 13R alpha1  [ **IL13RA1** ] , but not IL 4R alpha, were markedly decreased in in vitro monocyte derived macrophages MDMac, and with increasing time of monocytes in culture correlated with the loss of IL 13  [ **IL13** ]  regulation of lipopolysaccharide induced TNF alpha production.  Ref: 10427971 Eur J Immunol, 1999
- These receptors are composed of IL 13  [ **IL13** ]  receptor IL 13R alpha1  [ **IL13RA1** ] , IL 13Ralpha2, and IL 4Ralpha chains.  Ref: 11939409 Oncol Res, 2000
- **Il13ra1**  mRNA was co expressed with I14ra mRNA in mouse myeloid and natural killer cells on which IL 13  [ **IL13** ]  has been known to act.  Ref: 11003391 Immunogenetics, 2000
- We determined the allele frequencies of five polymorphisms in the  **IL13** ,  **IL13RA1** , and IL4RA genes in 588 continuing smokers chosen from the NHLBI Lung Health Study for having the fastest n = 282 and slowest n = 306 5 yr rate of decline of lung function mean change in FEV 1 %predicted yr = 4.1 and 1.1, respectively.  Ref: 12594065 Am J Respir Cell Mol Biol, 2003
- Polymorphisms in the  **IL13** ,  **IL13RA1** , and IL4RA genes and rate of decline in lung function in smokers.  Ref: 12594065 Am J Respir Cell Mol Biol, 2003
- IL 13  [ **IL13** ]  and the IL 13  [ **IL13** ]  specific receptor chain IL 13R alpha1  [ **IL13RA1** ]  are frequently expressed in cHL derived cell lines and in RS cells from biopsies of cHL tissues.  Ref: 12152987 Leuk Lymphoma, 2002
